# Student adjustment during Covid-19 pandemic: Exploring the moderating role of university support

**DOI:** 10.1016/j.heliyon.2023.e13625

**Published:** 2023-02-11

**Authors:** Edem M. Azila-Gbettor, Leonard Agbenyo, Hellen M. Fiati, Christopher Mensah

**Affiliations:** a Department of Management Sciences, Ho Technical University, Ghana; b Department of Logistics and Supply Chain Management, Ho Technical University, Ghana; c Department of Hospitality and Tourism Management, Ho Technical University, Ghana

**Keywords:** Fear of Covid-19, Academic adjustment, Psychological adjustment, Social adjustment

## Abstract

The study investigates the moderating effect of university support on the association between fear of Cov19 and student adjustment including (a) academic; (b) psychological; and (c) social adjustment. A total of 592 students took part in the research by completing self-reported questionnaire. The respondents were chosen using a multi-stage sampling technique. The data was processed and analyzed using IBM SPSS version 24 and SEM-PLS, respectively. Results reveal fear of Cov19 negatively predict student's academic, psychological, and social adjustment. University support positively predicts student's academic, psychological, and social adjustment and further mitigate the impact of fear of Cov19 on students' academic, psychological, and social adjustment. This is the first study to examine university support as a moderator between fear of Cov19 and three dimensions of students' adjustment.

## 1 Introduction

The novel coronavirus disease (CoV19) is a contagious respiratory and vascular disease caused by severe acute respiratory syndrome (SARS). CoV19 is the seventh member of the coronavirus family to affect humanity [1]. Since its emergence in China in late December 2019, the CoV19 virus has evolved and spread rapidly across the globe [2], compelling the World Health Organization (WHO) to declare the outbreak a pandemic [3]. Undoubtedly, the detrimental and ripple effect of CoV19 on every aspect of human life is confirmed in literature [4,5]. At the peak of the pandemic, CoV19 was estimated to have infected 25 million individuals, resulting in 840 000 deaths globally, representing a case fatality rate of 3.4% as of August 31, 2020 [3]. In the aviation sector, the International Air Transport Association (IATA) estimates global demand for air travel to decline significantly, resulting in US$113 billion in revenue losses [6]. CoV19 is reported may jeopardize the process of implementing the sustainable development goals due to the expected global recession [7,8] and rise in global poverty [9]. In the educational sector, close to 1.6 billion students and 63 million educators in 190 countries as of August 2020 were affected by the closure of schools across all levels of education [10]. Several authors [[11], [12], [13]] reported that universities across the globe responded to CoV19 in different ways, either by using face-to-face teaching with social distancing, deferring all academic activities, or deploying technology in the delivery of their programmes. The change in format of delivery of education has been found to impact learning pedagogy [14], resulting in an increase in the cost of education [15], actual learning losses [16], and lower levels of satisfaction with academic life among students [17].

One area of research that academics have pursued following the emergence of CoV19 is the “fear of CoV19” due to its negative effect on all aspects of human life [18,19]. de Hoog et al. [20] define fear as an unpleasant emotional state brought on by the perception of threatening stimuli. Fear is a sign that individuals are concerned about maintaining their health or wellbeing and further functions a driving force in engaging in protective behaviours [21]. Fear has been reported to generate emotional issues such as anxiety and stress disorders, and responsible for mental health impairment [22,23]. Evidence suggests that students who are exposed to the danger of infection may have concerns about transmission, worry about their health, and a dread of infecting others or dying [24,25]. Stress and anxiety are negative consequences of mental fatigue caused by adherence to social distancing, lockdowns and quarantines [26]. Additionally, the fear of Cov19 has triggered social exclusion and suicidal thoughts among students [27,28].

In educational studies, students’ adjustment to their complex environment has been found to be a useful concept [29,30]. For example, student adjustment is considered a significant determinant of their emotional, psychological, and relational wellbeing [31,32] and overall academic success [33]. Conversely, poor adjustment among students is associated with academic failure, antisocial conduct, and school dropout [34]. Given the importance of adjustment to students, it is imperative for academics to understand how the fear of CoV19 impacts the adjustment of students. Available data suggest that while studies student adjustment generally in Africa is highly concentrated in Southern Africa region [35,36], there is also a paucity of studies on the specific dimensions such as academic, social and psychological adjustments. Besides, a review of existing studies suggests that while empirical studies on fear of CoV19 have received considerable attention [[37], [38], [39], [40]] contend that the scientific literature is heavily tilted towards clinical effects compared to psychosocial effects. Furthermore, the link between fear of CoV19 and student adjustment, especially, academic, psychological, and social dimensions of adjustment, remains unexplored in the higher education setting. Karakose et al. [40] argue that the less attention paid to the psychological component is worrying if the world is to win the fight against the pandemic. Consequently, a study of the relationships between fear of CoV19 and student adjustment is significant because it will encourage the development and use of unique policy interventions that address students' psychological concerns.

In another vein, support in any form has been described as vital to students' education [41,42]. For example, perceived university support has been found to directly and indirectly influence several outcome variables among students [43,44]. Specifically, support provided by universities may help in lessening the negative effect of CoV19 on students, improve their wellbeing [45] and limit their attrition rates [46]. In spite of the significant contribution of university support, there is a paucity of research investigating university support as a moderator in the link between fear of CoV19 and student adjustment. This study proposes to examine university support as a boundary condition in the relationship between the fear of CoV19 and student adjustment. This study contends that university support may be an excellent medium for reducing the negative effect of fear of CoV19 on students’ adjustment. Consequently, the purpose of the study is to investigate the moderating effect of university support on the relationship between fear of CoV19 and student adjustment. More specifically, the study aims to explore the following objectives.

1. Assess the effect of fear of CoV19 on students' *(a)* academic; *(b)* psychological; and *(c)* social adjustment.
2. Determine the effect of university support on students *(a)* academic; *(b)* psychological; and *(c)* social adjustment.
3. Assess the moderating effect of university support on the relationship between fear of CoV19 and *(a)* academic; *(b)* psychological; and *(c)* social adjustment.

The study contributes to the CoV19 literature in the following ways: First, we expand on the existing CoV19literature by investigating its effects on students’ adjustment. Furthermore, the study examines university support as boundary conditioning in mitigating the negative effect of fear of CoV19 on the academic, psychological, and social adjustment of students. Lastly, we enrich existing literature from underrepresented contexts.

The remainder of the paper is structured as follows: Section 2 describes the literature review for the study. The method of the study is presented in Section 3. The results of the study are presented in Section 4. Section 5 covers discussions and the theoretical and empirical implications of the study. In the last part, the study's limitations and future research directions are discussed, as well as the study's conclusion.

## 2 Literature review

### 2.1 Fear of CoV19

Even though “fear” is the most widely researched emotion [47], existing literature points to a significant variation in its definition across several disciplines [48]. Ashkanasy and Nicholson [49] proposed a comprehensive conceptualization of fear as “a generalised experience of apprehension” (p. 24). According to de Hoog et al. [2], fear is an unpleasant emotional state resulting from the perception of threatening stimuli. In the psychological sciences, fear is defined as, “a reaction to an external stimulus … and appears to be associated with autonomic hyper-arousal when the individual is exposed to the stimulus … a common adaptive response to an immediate, threatening situation” [50]. Fear has been the most prevalent emotional reaction among the public following the emergence and spread of the CoV19 epidemic [51]. Since the emergence of CoV19 in late 2019, there has been an increasing level of fear among the public due to the deadly impact the virus has on human life. Generally, this fear has resulted in high levels of anxiety, stress, and depression [52,53]. Regarding students, Elsharkawy and Abdelaziz [54] found fear of CoV19 to be high among females and students with family members who had contracted or been exposed to the virus. Several studies have found fear of CoV19 to be associated with symptoms of fear, anxiety, depression, mental health distress, interpersonal disengagement, struggles with motivation, boredom, anxiety, depression, and sleep disorders among students [[55], [56], [57]]. However, female students most often than not suffer from additional stressful life events during the pandemic than their male counterparts [58,59]. References [56,60] found fear of CoV19 among students to have resulted in greater fear of colleagues within their social network, social isolation, and academic-related pain due to the radical change from conversional methods of learning to online learning. Finally, another apprehension of fear of Cov19 among students is the fear of missing out on significant and meaningful social experiences [61,62], which reduce life satisfaction [63].

### 2.2 Student adjustment

Adjustment is the process of making accommodations to demands and restrictions including the freedom to live and work with others by engaging in satisfying interactions and relationships [64]. Ramsay et al. [65] refer to it as a “dynamic process” that might lead to the right fit between a person and their environment. Adjustment in the context of university students is considered a multifaceted and complex phenomenon [66]. In this study, student adjustment has been examined based on three dimensions namely psychological, social, and academic adjustment [[67], [68], [69]]. Psychological adjustment defines a person's subjective sense of distress and the extent to which one functions in normal life [70,71]. Academic adjustment defines a student's positive attitude and valuation towards their academic work and environment [72]. Social adjustment describes an individual's ability to adapt to their social setting by engaging in appropriate and effective social interaction [73,74]. Generally, the adjustment of students has been found to support student academic success and social life [75]. Individuals with a high level of psychological adjustment possess the ability to work normally during crises [71]. High positive psychological adjustment has been linked to increasing satisfaction and quality of a student's life while reducing impediments associated with student depression, anxiety, stress and burnout [71,76]. In a recent study, Okado et al. [77] examined 228 college and university students in the USA and reported a significant increase in depression, and anxiety during CoV19 due to the absence of psychosocial support. Prior studies have shown that the social and academic adjustment of students is associated with effective learning and improved academic achievement [[78], [79], [80]].

### 2.3 Fear of CoV 19 and student adjustment

Aydin et al. [81] posits that people's responses to adverse living conditions reflect their adjustment skills. The fear of CoV19 is suggested in the literature as a negative predictor of all forms of student adjustment [52]. A growing body of studies suggests that fear of CoV19 poses a considerable concern for its potential to upset individuals' psychological adjustment [82]. For example, Seçer et al. [82] found support for a negative association between fear of CoV19 and the psychological adjustment of 390 healthcare professionals in Turkey. In another study, Arslan et al. [68] found that fear of CoV19 negatively predicted the psychological adjustment of 315 undergraduate students in Turkey. Panchal and Yadav [69] also studied 136 respondents and found fear of CoV19 to negatively affect their emotional adjustment. In a recent systematic review, Branje and Morris [83] found that fear of CoV19 among adolescent students negatively affected their academic and social adjustment. Finally, the fear of CoV19 including anxiety and depression was found to be negatively associated with students' academic adjustment [68,84,85] and social adjustment [86]. The studies reviewed above convincingly support the notion that students who entertain fear have unfavourable social, intellectual, and psychological adjustments. Consequently, we hypothesized that:

H1a Fear of CoV19 negatively predict students' academic adjustment.

H1b Fear of CoV19 negatively predict students' psychological adjustment.

H1c Fear of CoV19 negatively predict students' social adjustment.

### 2.4 University support and student adjustment

The scope of support services offered by higher educational institutions to students is vast and different [87]. Generally, university support describes the support system that encourages stakeholders, including teachers and students, to undertake their core mandate in a highly efficient and productive way [88]. Institutions provide an array of support services that may include academic advising, career and personal counselling, and financial assistance, among several others, to address the needs of students [89]. Support offered by institutions has been shown to protect students from psychological distress [90]. Similarly, Hill et al*.* [91] found student support systems to be a significant factor in fostering a sense of belonging and the delivery of quality education among higher education students. Cutrona and Russell [92] posited that a failed or successful adaptation to a higher educational environment is determined by the availability and type of support services in the institution. Several studies have found support as an empowering factor in enabling students to meet their university challenges [[93], [94], [95]]. In a recent study conducted by Martinez-Lopez [95], social support was found to predict adjustment among 300 first year students. Similarly, Van Gorp et al. [96] found support as a positive coping mechanism for adjusting psychologically to stressful conditions. Finally, a strong link was found between university support and social adjustment [97]. Consistent with earlier studies, we expected that university support would reliably predict the adjustment of university students. Specifically, we expect university support to positively influence the social, psychological, and academic adjustment of students. Consequently, we hypothesized that:

H2a *University support positively predict students' academic adjustment.*

H2b University support *positively predict students' psychological adjustment.*

H2c *University support positively predict students' social adjustment.*

### 2.5 Moderating role of university support

Previous studies have given credence to “support” as a significant resource and found it to play a moderating role in the nexus between personal and organizational factors and outcomes [98]. For example, Noh and Kaspar [99] found social support to heavily influence the coping effectiveness of Korean immigrants in Toronto. Similarly, Ng et al. [100] discovered that support received by Chinese university students significantly moderated the effect of acculturation strategies on cultural adaptation. In the context of the CoV19 pandemic, Ullah et al*.* [101] suggest that one way students can adjust is through support systems that protect their psychological, physical, and emotional wellbeing. We expect that students' access to university support has the potential to minimize their fear of CoV19 while increasing the level of academic, psychological and emotional adjustment. It can therefore be concluded that university support is an important moderator in explaining the relationships between fear of CoV19 and students’ academic, social and psychological adjustment. Based on the above evidence, we hypothesise that.

H3a University support will positively moderate the relationship between fear of CoV19 and students' academic adjustment.

H3b University support will positively moderate the relationship between fear of CoV19 and students' psychological adjustment.

H3c University support will positively moderate the relationship between fear CoV19 and students' emotional adjustment.

**Fig. 1:**
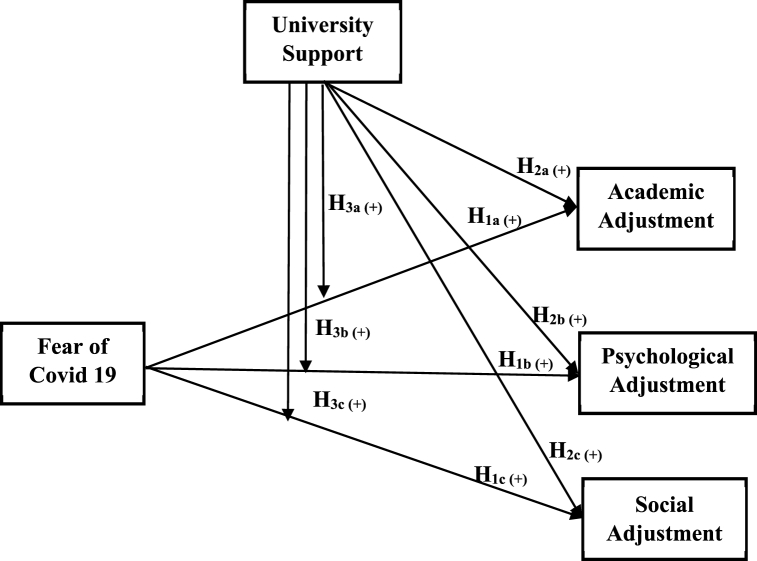
Moderating model of University Support, Fear of Covid 19 and Student adjustment.

The model below illustrates the expected relationship between the three variables (Fig. 1).

## 3 Methods

### 3.1 Procedure and participants

The study's target population consisted of undergraduates at a Technical University in Ghana. Based on a population size of 8,500, the sample size was calculated to be 573 [102]. However, to attain a large response rate, a 750 sample was used for the study. Six hundred and seventy-three (673) questionnaires were returned by the research participants, and 592 were found to contain relevant information and were used for the study. The respondents were chosen using a multi-stage sampling approach. In the first stage, a stratified sampling technique was used to enable proportionate representation of all departments [103]. The actual samples were drawn from each stratum (department) using a simple random sampling method. The sampling frame was created using the students' registration list obtained from the institution's registry. The data for the study was collected between July–September 2020. All authors participated in the collection of the data. Prior to data collection, respondents were informed of their voluntary participation and withdrawal. They were further guaranteed anonymity and confidentiality of the information provided. The study complied with the standards and principles of the Declaration of Helsinki for research on human beings, and it received approval from the university's Research and Ethics Review Committee.

The profile of the sample is presented in Table 1. Of the 592 questionnaires retrieved, females constituted 51.5% of the sample, while 48.5% were males. The most dominant age group was 21–25 years and 86.6% of the respondents were unmarried. Majority of the respondents (35.6%) were in their second year. Respondents' demographic breakdown is reflective of the profile of Ghanaian university students [[104], [105], [106], [107], [108], [109], [110]].

**Table 1:** Profile of respondents.

	Characteristics	Frequency	Percentage
Gender	Male	287	48.5
	Female	305	51.5
Age	≤20 yrs.	131	22.1
	21–25 yrs.	319	53.9
	≥26 yrs.	142	24.0
Marital Status	Married	79	13.4
	Single	513	86.6
Year of Study	1st year	198	33.4
	2nd year	211	35.6
	3rd Year	183	30.9

### 3.2 Instrumentation/data collection

The survey used for the study was divided into two sections. In Section A, we asked respondents to provide information on their personal profiles, such as their age, marital status, gender, year of study, and age. Section B covers information on the constructs examined in the study such as FCov19, and student adjustment including academic adjustment, psychological adjustment, and social adjustment. A total of 38 items were selected from the pool of self-reported measures that had been validated (Table 2). The original versions of each scale were written in English.

**Table 2:** Sources of measures of concepts.

Latent Construct	Source	No. of Items	Range of Scale
**Fear of Covid-19**	[24]	7	5-point; **1**(S*trongly disagree) to***5**(S*trongly agree)*.
**University support**	[111]	*10*	5-point; **1**(S*trongly disagree) to***5**(S*trongly agree)*.
**Psychological adjustment**	[112]	4	5-point; **1**(S*trongly disagree) to***5**(S*trongly agree)*.
**Academic adjustment**	[112]	6	5-point; **1**(S*trongly disagree) to***5**(S*trongly agree)*.
**Social adjustment**	[112]	9	5-point; **1**(S*trongly disagree) to***5**(S*trongly agree)*.

Sample items from variables used include (i) university support- “The University provides a clear instructional guide for CoV19 .; (ii) **FCov19**- “It makes me uncomfortable to think about covid-19”; (iii) **academic adjustment**- “adjusting to academic standards or expectations has been difficult during the CoV19 era”; (iv) **social adjustment -**adjustment to the social environment has been difficult in the university during the CoV19 era”; and (v) **psychological adjustment -** “I felt insecure going back to school during the CoV19 era.”.

### 3.3 Analytical approach

The characteristics of respondents were analyzed using frequency and percentages. The proposed hypotheses were analyzed using PLS-SEM. Using the work of Hair et al. [113] as a guide, the PLS algorithm was used to evaluate the model's fit, discriminant validity, adjusted R^2^, and inner VIF values. Further, the structural model of the study was evaluated using bootstrapping sampling (10 000 re-samples) to examine the coefficients and significant levels of the paths. Results of the Common Method Bias estimate using Kock's [114] approach revealed that all the factor levels are less than 3.3 (Table 3). Hence, the model is free of common method bias.

**Table 3:** Factor loadings, validity and reliability of latent constructs.

Constructs and Items	Loadings	VIF	CR	CA	AVE
*Fear of Covid-19 (FCov19)*			0.861	0.799	0.554
**FCov19**_**1**_	0.760	1.628			
**FCov19**_**2**_	0.785	1.719			
**FCov19**_**3**_	0.738	1.492			
**FCov19**_**4**_	0.735	1.531			
**FCov19**_**5**_	0.703	1.423			
*University Support (US)*			0.797	0.717	0.568
**US**_**1**_	0.759	1.266			
**US**_**2**_	0.813	1.343			
**US**_**3**_	0.783	1.153			
**US**_**4**_	0.768	1.276			
**US**_**5**_	0.814	1.286			
**US**_**7**_	0.722	1.911			
**US**_**8**_	0.871	1.941			
**US**_**9**_	0.743	1.315			
**US**_**10**_	0.838	1.243			
*Psychological Adjustment (PsyA)*			0.826	0.721	0.546
**PsyA**_**1**_	0.790	1.501			
**PsyA**_**2**_	0.802	1.555			
**PsyA**_**3**_	0.742	1.390			
**PsyA**_**4**_	0.742	1.521			
*Academic Adjustment (AA)*			0.791	0.749	0.658
**AA**_**1**_	0.713	1.124			
**AA**_**2**_	0.899	1.114			
**AA**_**3**_	0.733	1.126			
*Social Adjustment (SA)*					
**SA**_**1**_	0.856	1.364	0.862	0761	0.758
**SA**_**2**_	0.885	1.364			
**SA**_**3**_	0.743	1.374			

**Notes:** FL, factor loading; a, Cronbach's Alpha; CR, composite reliability; AVE, average variance extracted.

## 4 Results

### 4.1 Measurement model assessment

The measurement model's quality was determined by the validity and reliability of latent construct coefficients. The model was considered appropriate for structural analysis based on the results of the latent constructs [113] (Table 3). For example, Composite Reliability (CR) coefficients ranged from 0.791 to 0.862, above the specified limit value of 0.70 [115]. Furthermore, Cronbach alpha (CA) values varied from 0.717 to 0.799, above the suggested minimum of 0.7 [116]. Furthermore, the coefficients of Average Variance Extracted (AVEs) for all variables exceeded 0.50, ranging from 0.546 to 0.758, indicating that the model's latent variables are valid and reliable [117].

The model's discriminant validity was evaluated using Fornell-Larcker [118] and Heterotrait-Monotrait criteria [119]. As reported in Table 4, the square root of the AVEs of all constructs in the matrix diagonal is greater than the associated correlations in the corresponding columns and rows, thus demonstrating the quality of the reflective model [120]. For instance, the AVE for FCov19 (0.745) is greater than the corresponding row correlation (0.364) and column correlation (0.594, 0.163, 0.350). Accordingly, the three latent variables used in the research model are different, thus suggesting the quality of the measured construct. In respect of the HTMT criterion for assessing discriminant validity, all the correlations (Table 5) were lower than the suggested limit of 0.90 [119,121,122], demonstrating that the three latent constructs used in the research were conceptually different.

**Table 4:** Fornell-larcker criterion.

	AA	FCov19	PsyA	US
**Academic adjustment** (AA)	0.811			
**Fear of Covid-19** (FCov19)	0.364	0.745		
**Psychological adjustment** (PsyA)	0.434	0.594	0.739	
**Social adjustment** (SA)	0.025	0.112	0.150	0.871
**University support** (US)	0.205	0.350	0.450	0.285

**Table 5:** Heterotrait-monotrait ratio (HTMT).

	AA	FCov19	PsyA	US
**Academic adjustment** (AA)				
**Fear of Covid-19** (FCov19)	0.552			
**Psychological adjustment** (PsyA)	0.747	0.772		
**Social adjustment** (SA)	0.075	0.163	0.218	
**University support** (US)	0.357	0.505	0.672	

### 4.2 Model estimation

The model fit was assessed using the standard root mean square residual (SMSR) value [123] The SRMR of the model was 0.079 < 0.08, demonstrating a good model fit [124] (Table 5). The models' explanatory power was assessed using the adjusted *R*^2^ criterion [125]. The findings regarding R^2^ indicate FCov19, and university support explains 23.5%, 41.7% and 37.6% of the variations in students' academic, psychological, and social adjustments, respectively. The model's predictive validity was assessed using Stone-Geisser's *Q*^2^ Test [126,127]. The Q^2^ values of academic adjustment (0.102), psychological adjustment (0.234) and social adjustment (0.170) demonstrate medium and small predictive relevance [128] (Table 6). Cohen's [129] *f*^*2*^ was used to assess the effect size of the main exogenous construct. Analysis of results suggests the magnitude of the effect of fear of Cov19 on academic adjustment (*f*^*2*^ = 0.113), psychological adjustment (*f*^*2*^ = 0.375) and social adjustment (*f*^*2*^ = 0.102) met the effect threshold of medium effect size. Similarly, the magnitude of the university's support for academic adjustment (*f*^*2*^ = 0.108), psychological adjustment (*f*^*2*^ = 0.114) and social adjustment (*f*^*2*^ = 0.105) met the effect threshold of medium effect size.

**Table 6:** Summary of fit and R^2^ of structural model.

Construct Coefficient of Determination (R^2^)	R^2^	Adjusted R^2^	Q^2^
**Academic adjustment**	0.239	0.235	0.102
**Psychological adjustment**	0.420	0.417	0.234
**Social adjustment**	0.381	0.376	0.170
Model Fit	**Value**		
**SRMR**	0.079		

The collinearity among the independent constructs was assessed using VIF prior to testing the hypotheses [130]. Findings in Table 7 show that the VIFs values of a pair of FCov19 and university support are below 3, indicating that there is no correlation between the determinants of students' academic, social, and psychological adaptations. The results of the direct (H_1a_-H_2c_) and indirect (H_3a_-H_3c_) hypotheses are shown in Table 8.

**Table 7:** **Collinearity assessment** (**inner VIF values)**.

	AA	FCov19	PsyA	US
**Academic adjustment** (AA)				
**Fear of Covid-19** (FCov19)	1.140		1.140	
**Psychological adjustment** (PsyA)				
**Social adjustment** (SA)				
**University support** (US)	1.140		1.140

**Table 8:** Path coefficient and hypothesis assessment of direct and indirect paths.

Hypothesis	Path	Path Coefficient	T Statistics	P Values
H_1a_	Fear of Covid 19 - > Academic adjustment	−0.354	6.919	0.000
H_1b_	Fear of Covid 19 - > Psychological adjustment	−0.516	12.529	0.000
H_1c_	Fear of Covid 19 - > Social adjustment	−0.237	4.638	0.031
H_2a_	University support - > Academic adjustment	0.166	2.242	0.021
H_2b_	University support - > Psychological adjustment	0.256	5.782	0.000
H_2c_	University support - > Social adjustment	0.255	4.934	0.000
	*Moderating effect of university support on*			
H_3a_	Fear of Covid 19 - > Academic adjustment	0.136	3.584	0.000
H_3b_	Fear of Covid 19 - > Psychological adjustment	0.164	3.618	0.000
H_3c_	Fear of Covid 19 - > Social adjustment	0.171	3.785	0.000

P Values Significant @ 95%.

The relationship between FCov19 and academic adjustment is shown to be negative and significant, supporting **H**_**1a**_ (*ß* = −0.354; *t* = 6.919; *p* = 0.000). This result suggests students’ levels of academic adjustment are lessened with the persistent fear of Cov19.

The relationship between FCov19 and psychological adjustment is confirmed to be negative and significant, supporting **H**_**1b**_ (*ß* = −0.516, *t* = 12.529, *p* = 0.000). The finding suggests students who are reported to be afraid of Cov19 suffer from psychologically adjusting to their educational environment.

In support of **H**_**1c**_**,** the relationship between FCov19 and social adjustment is negative and statistically significant (*ß* = −0.237; *t* = 4.638; p = 0.031). The finding reveals that students’ levels of social adjustment are negatively impacted by the fear of Cov19.

The relationship between university support and academic adjustment is positive and statistically significant (*β* = 0.166; *t* = 2.242; p = 0.021), confirming **H**_**2a**_*.*The finding suggests that university support enhances students’ levels of academic adjustment.

The relationship between university support and psychological adjustment is positive and statistically significant (*β* = 0.256; *t* = 5.78; *p* = 0.000), confirming **H**_**2b**_. The findings imply that it is possible for students to successfully adjust psychologically, provided university administrators provide the necessary support for learning.

The relationship between university support and social adjustment was positive and statistically significant (*β* = 0.255; t = 4.93; p = 0.000), confirming **H**_**2c**_*.*The finding shows social adjustment among students is attainable when support from the university is provided.

In support of **H**_**3a**_, the relationship between FCov19 and academic adjustment is moderated positively by university support (β = 0.136; *t* = 3.584; p = 0.000). This suggests students are likely to adjust academically in the face of persistent fear of Cov19 when there is enough support from the university.

In support of **H**_**3b**_, the relationship between FCov19 and psychological adjustment is moderated positively by university support (β = 0.164; t = 3.618; p = 0.000). This suggests the negative relationship between fear of Cov19 and psychological adjustment among students is improved when there is sufficient university support.

In support of **H**_**3c**_ the relationship between FCov19 and social adjustment is moderated positively by university support (β = 0.171; t = 3.785; p = 0.000). This suggests students are likely to adjust socially in the face of persistent fear of Cov19 when there is enough support from the university.

## 5 Discussions

The study investigates the relations between FCov19, university support, and student adjustment, i.e., psychological, academic and social adjustment amongst 592 students in a higher education setting. The structural model was validated, and the hypotheses presented were tested using a partial least squares structural equation model.

Consistent with earlier studies, FCov19 negatively predicted academic, psychological, and social adjustments [52,82,83], thereby supporting hypotheses H_1a_, H_1b_ and H_1c_. The findings affirm the negative consequences of FCov19, suggesting that FCov19 has inflicted an immense emotional, psychological, and academic burden on students [[131[, [132], [133]]. The result suggests that students who experience FCov19 are more likely to have difficulties with academic, psychological, and social adjustments compared to those with less or no fear of Cov19. This phenomenon of maladjustment due to FCov19 may intensify students' feelings of uncertainty and anxiety [134]. This may likely impede students' ability to concentrate during lectures and their private learning sections [135] and their general learning process [136,137]. The evidence highlights the need for university management and instructors, to be mindful of any change in students’ social, psychological and social behavioural tendencies, no matter how subtle or reserved the students may be.

University support was found to positively predict students' academic, psychological and social adjustments. This finding corroborates earlier work [[93], [94], [95]] and supports hypotheses H_2a_, H_2b_ and H_2c_. The study extends the discourse by exploring perceived university support as a moderator in the relationship between FCov19 and students' academic, psychological, and emotional adjustments. Perceived university support moderates the relationship between FCov19 and student adjustment, that is, academic, social, and psychological adjustment, thereby supporting hypotheses H_3a_, H_3b_ and H_3c_. More specifically, the findings suggest support provided by the university enhances students’ social, academic, and psychological adjustment during the period of Cov19. Furthermore, the results validate the positive effect of university support on the negative relation between FCov19 and student adjustment. The positive relationship between university support and the dimensions of student adjustment, as well as, the moderating effects, is not surprising, especially in Africa, since most higher learning institutions have adopted multifaceted approach and response in dealing with the pandemic. The supportive environment provided by the university represents an extension of personal resource [65] and further serves as a protective factor in mitigating the negative effect of FCov19 [138,139] on their level of academic, social, and psychological adjustments.

## 6 Implications for theory and practice

The study contributes to existing theory by proposing a moderating mechanism to examine the effect of FCov19 on students' psychological, academic, and social adjustments which remain unexplored in the higher education literature. The findings validate the direct hypothesis that increasing FCov19 has a negative effect on students' level of adjustment. The findings further support a positive effect of university support on the fear of Cov19 on the level of students’ adjustment.

The findings suggest higher education institutions should pay careful attention to developing support structures to enhance the efficient adjustment of students in the face of a perceived threat. Siebenhaar et al. [140] posit that people who have adequate and appropriate information about Cov19 experience less stress. Therefore, to soothe students' anxieties, university authorities must set up training programmes on CoV19 and use a variety of video conferencing and social media platforms to propagate educational information. By doing so, students will be better able to adjust to their current environment, enhance their wellbeing, and reduce their chances of experiencing other psychological difficulties in the near future. University authorities should also launch an educational campaign to promote health messages that will prevent the spread of CoV19 and encourage students to report any symptoms of the disease to health professionals. In the area of support, universities must adopt a collaborative approach by working with students’ associations or unions and other networks to present innovative and engaging programmes of virtual activities. Additionally, authorities must use the services of school counsellors to calm the nerves of students by providing the necessary emotional and psychological support.

## 7 Limitations and future research directions

The following limitations should be considered when interpreting the findings of the study: Firstly, the study's use of self-reported data based on respondent subjective perception. These raise concerns about the probability of common technique variance (CMV) [141]. However, an assessment of factor-level VIFs, using the Kock's [114] approach indicated the absence of CMV. Secondly, the study adopted a cross-sectional design. That all constructs were measured at a point in time. The testing of causality between the variables would be hampered by this technique. Therefore, longitudinal research designs should be given consideration in future studies.

The study's findings point to several potential research paths in the future. Replication of the research framework in other higher educational settings is highly recommended. Future studies can also consider examining other dimensions of support, such as lecturer, social, peer, and parents as moderators in the association between FCoV19 and student adjustment. Similarly, future studies may consider examining students' personal resources, such as hope, resilience, and optimism as a moderator in the proposed relationship. Future studies can expand the model into a mediation moderated model by identifying and introducing mediators through a vigorous literature search.

## 8 Conclusions

This study examines three broad research objectives in the process of examining the moderating effect of university support on the direct relationship between FCoV19 and student adjustment. A total of 592 students was used to validate a set of nine hypotheses. The finding suggests students’ levels of academic, psychological, and social adjustment are lessened with the persistent fear of CoV19. The findings further suggest that students are likely to adjust academically, psychologically, and socially in the face of persistent fear of Cov19 when the university provides adequate support mechanisms.

## Author contribution statement

Edem M. Azila-Gbettor: Conceived and designed the research; Analyzed and interpreted the data; Wrote the paper. Christopher Mensah: Conceived and designed the research; Contributed reagents, materials, analysis tools or data; Wrote the paper. Leonard Agbenyo; Hellen Fiati: Contributed reagents, materials, analysis tools or data; Wrote the paper.

## Funding statement

This research did not receive any specific grant from funding agencies in the public, commercial, or not-for-profit sectors.

## Data availability statement

Data will be made available on request.

## Declaration of competing interest

The authors declare no competing interests.
